# Hand eczema and lifestyle factors in the Dutch general population: Evidence for smoking, chronic stress, and obesity

**DOI:** 10.1111/cod.14005

**Published:** 2021-12-05

**Authors:** Laura Loman, Marie L. A. Schuttelaar

**Affiliations:** ^1^ Department of Dermatology University of Groningen, University Medical Center Groningen Groningen The Netherlands

**Keywords:** general population, hand dermatitis, hand eczema, lifestyle factors, obesity, smoking, stress

## Abstract

**Background:**

Several risk factors, among other lifestyle factors, have been suggested for hand eczema (HE).

**Objectives:**

To investigate a possible association between HE and lifestyle factors, including smoking, alcohol consumption, stress, body mass index (BMI), waist circumference, physical activity, diet, and amount of sleep in the Dutch general population.

**Methods:**

Data from the large population‐based LifeLines Cohort Study was used. Individuals with HE in the past year were identified by a cross‐sectional questionnaire in 2020. At baseline, information on lifestyle factors was collected.

**Results:**

In total 57 046 individuals were included in the present analysis. Smoking ≥8 cigarettes/day, and smoking ≥15 pack years showed a positive association with HE in the past year. In addition, chronic stress, a BMI >30 kg/m^2^, and a waist circumference of >90 cm were positively associated with HE in the past year.

**Conclusions:**

The current study indicates that lifestyle factors are associated with HE. Advice regarding lifestyle factors might contribute to enhance overall health, of which HE might possibly benefit in conjunction. Further studies should also focus on the association between lifestyle factors and the severity and prognosis of HE rather than on occurrence alone.

## 1 INTRODUCTION

Hand eczema (HE) is an inflammatory skin disease with a 1‐year prevalence of 9.1% in the general population.^1^, ^2^ It may cause both far‐reaching personal consequences, with an impaired quality of life of those affected, and socio‐economic consequences in terms of sick leave, job‐loss and change, and high health care costs.^3^, ^4^, ^5^ The pathogenesis of HE has not yet been fully elucidated, but both endogenous and exogenous factors are assumed to play a role.^6^ Several risk factors, such as atopic dermatitis (AD),^7^ contact allergy,^8^ and wet work,^9^, ^10^ are known to cause or contribute to HE.

The association between dermatological diseases and lifestyle is increasingly a subject of research. Diverse lifestyle factors can influence the immune system and alter inflammatory processes. Therefore, it is hypothesized that when improving overall health, HE might benefit in conjunction. Lifestyle and behavioral changes might be of great importance in future complementary medicine, with a possible role for prevention and personalized treatment programs for HE. Tobacco smoking has been the most extensively studied in HE, but results are pointing in different directions.^11^, ^12^ In addition, evidence of the role of other lifestyle factors in HE is scanty.

The aim of the present study was to investigate a possible association between self‐reported HE in the past year and several lifestyle factors in the Dutch general population.

## 2 MATERIALS AND METHODS

### 2.1 Study population and design

Data was obtained from the LifeLines Cohort Study, which has been described previously.^13^ Briefly, The LifeLines Study is a multi‐disciplinary prospective population‐based cohort study, examining the health and health‐related behaviors of 169 729 persons living in the North of The Netherlands in a unique three‐generation design. It employs a broad range of investigative procedures in assessing the biomedical, sociodemographic, behavioral, physical, and psychological factors that contribute to the health and disease of the general population, with a special focus on multi‐morbidity and complex genetics. All procedures were approved by the Medical Ethics Committee of the University Medical Center Groningen (reference number: METc 2007/152, and METc 2019/571) and written informed consent was given by the participants.

For the current study, data from the self‐administrable digital add‐on questionnaire regarding skin diseases, sent out in 2020, were used. The questionnaire was sent to 135 950 adult LifeLines participants, of which 58 198 participants (42.8%) responded. Data regarding the prevalence and severity of HE for this study population has been described previously.^14^ Of the responders, 57 046 were 18 years or older at baseline, responded to the question regarding lifetime prevalence of HE, and were used for further analysis in the current study.

### 2.2 Questionnaire

The lifetime prevalence of HE was identified by the question “Have you ever (now or in the past) had hand eczema?” and HE in the past year was identified by the question “Have you had hand eczema in the past 12 months?” (both based on the Nordic Occupational Skin Questionnaire [NOSQ‐2002]; Q.D1).^15^ Subjects with HE in the past year were compared with subjects who never had HE. Variables regarding HE, AD, and exposure to wet activities were included in the add‐on questionnaire. Data on lifestyle factors were extracted from the baseline assessment of the LifeLines Cohort Study, collected between 2006 and 2013. (See online supplementary material S1 for further details of all the questions.)

### 2.3 Lifestyle factors

#### 2.3.1 Smoking

Smoking behavior was categorized as never‐, former‐, and current smokers. Current smokers were categorized in smoking <8 and ≥8 cigarettes per day. One pack year was defined as smoking 20 cigarettes per day for 1 year; cigars were regarded as three cigarettes.

#### 2.3.2 Alcohol consumption

Alcohol consumption was categorized as non‐drinker, ≤1 alcoholic drink/day, >1‐2 alcoholic drinks/day, and >2 alcoholic drinks/day.

#### 2.3.3 Stress

The occurrence of stressful life events and chronic stress experienced in the last 12 months were measured using the List of Threatening Experiences (LTE)^16^ and the Long‐term Difficulties Inventory (LDI),^17^ respectively. Both have been validated for large population‐based cohorts.^18^ The LTE compromises 12 life events, for which participants indicated whether or not the life events occurred, with a maximum total score of 12. Total scores were categorized in 0, 1, 2, and ≥3 points. The LDI consists of 12 life aspects for which participants indicate how they experienced these life aspects with respect to difficulty and stress on a three‐point scale: 0 = not stressful, 1 = slightly stressful, and 2 = very stressful. Total scores range from 0 to 24, and were categorized as 0, 1‐2, 3‐4, and ≥5 points, with higher scores indicating more stress.

#### 2.3.4 BMI

BMI was calculated as kilogram per square meter (kg/m^2^) and further categorized: “underweight” (<18.5 kg/m^2^), “normal weight” (18.5‐25.0 kg/m^2^), “overweight” (>25‐30.0 kg/m^2^), and “obesity” (>30 kg/m^2^).

#### 2.3.5 Waist circumference

Waist circumference was categorized as: ≤80, >80‐90, >90‐100, >100‐110, and >110 centimeter  (cm).

#### 2.3.6 Physical activity

Physical activity was measured using the Short Questionnaire to Assess Health‐enhancing physical activity (SQUASH), which includes questions regarding commute activity, physical activity at work, household activities, and leisure time activities (including sports) of an average week in the past months.^19^ Intensity was categorized in to light, moderate, or vigorous based on age‐specific Metabolic Equivalent Tasks (METs) derived from Ainsworth's compendium of physical activity combined with the self‐reported intensity of each activity.^20^ Outcomes were presented as moderate and vigorous physical activity (MVPA), vigorous physical activity (VPA), and their tertiles in minutes per week (min/wk).

#### 2.3.7 Diet

Diet was categorized as a vegetarian and/or vegan diet, and the overall diet quality was assessed by using the LifeLines Diet Score (LLDS), a tool based on the 2015 Dutch Dietary Guidelines.^21^ It consists of 12 food groups, including 9 food groups with proven positive health effects (vegetables, fruit, whole grain products, legumes and nuts, fish, oil and soft margarines, unsweetened diary, coffee, and tea) and 3 food groups with negative effects (red and processed meat, butter and hard margarines, and sugar‐sweetened beverages). Per food group, the intake in grams per 1000 kcal is categorized into quintiles, awarded 0 to 4 points (negative groups scored inversely) and summed. The total LLDS ranges from 0 to 48, with higher scores representing a higher diet quality. For the present study the quintiles of the total LLDS were used.

#### 2.3.8 Sleep

The total minutes of sleep per 24 hours categorized in to 5, 5‐7, 7‐9, and >9 hours/24 hours.

### 2.4 Statistical analyses

Analyses were performed using the Statistical Products and Service Solutions package version 25 (SPSS Inc., Chicago, IL, U.S.A.). All proportions were computed excluding missing answers. Differences between responders and nonresponders were assessed using an independent Student *t*‐test, Mann‐Whitney *U* test, or a chi‐square test. Univariate and multivariate logistic regression models were performed with HE in the past year vs never HE as the dependent variable, and sex, age, self‐reported physician diagnosed AD, exposure to wet activities, and all lifestyle factors as the independent variables. To verify a possible dose‐response relationship between lifestyle factors and HE, all categorized continuous variables were also entered as continuous variables in separate models. Associations were expressed as odds ratios (ORs) with 95% confidence intervals (CIs). *P*‐values of <.05 were considered to be statistically significant.

## 3 RESULTS

### 3.1 Study population

The lifetime prevalence of HE was 15.0% (95% confidence interval [CI] 14.7‐15.3) and the 1‐year prevalence of HE was 7.3% (95% CI 7.1‐7.5). There was an increased proportion of female responders compared to nonresponders (60.3% vs 57.2%, respectively), and older individuals were less likely to answer the questionnaire compared with younger individuals (mean age ± standard deviation [SD] 55.8 ± 12.2 vs 50.6 ± 12.3 years, respectively; age at the time of answering the add‐on questionnaire in 2020). Overall, responders reported a more favorable lifestyle at the baseline assessment with less current smoking, less stress, and more moderate and vigorous activity compared to nonresponders (see online supplementary material S2).

### 3.2 Lifestyle factors and hand eczema

Subjects with HE in the past year were more often female (70.5%) compared with subjects without HE ever (58.3%). The 1‐year prevalence of HE decreased with age. More subjects with HE in the past year than subjects without HE ever reported AD (33.7% vs 5.7%, respectively) and exposure to wet activities (33.1% vs 23.2%, respectively) (Table 1).

**TABLE 1:** Hand eczema in the past year in relation to lifestyle factors and potential confounders

	Total	HE (past year)	HE never	Crude OR (95% CI)	*P*‐value	Adjusted OR (95% CI) model 1^a^	*P*‐value	Adjusted OR (95% CI) model 2^b^	*P*‐value
	n (%)	n (%)	n (%)						
Total	57.046 (100)	4.158 (7.3)	48.496 (85.0)	—	—	—	—	—	—
Sex									
Male	22.650 (39.7)	1.228 (29.5)	20.223 (41.7)	1	—	1	—	1	—
Female	34.396 (60.3)	2.930 (70.5)	28.273 (58.3)	**1.71 (1.59‐1.83)**	**<.001**	**1.60 (1.50‐1.72)**	**<.001**	**1.34 (1.24‐1.45)**	**<.001**
Age (y)^c^									
25‐34	3.080 (5.4)	362 (11.8)	2.552 (82.9)	1	—	1	—	1	—
35‐44	7.151 (12.5)	821 (11.5)	5.782 (80.9)	1.00 (0.88‐1.14)	0.99	1.06 (0.93‐1.21)	0.36	1.13 (0.98‐1.30)	0.10
45‐54	14.704 (25.8)	1.240 (8.4)	12.212 (83.1)	**0.72 (0.63‐0.81)**	**<.001**	**0.76 (0.67‐0.86)**	**<.001**	**0.83 (0.72‐0.95)**	**0.006**
55‐64	18.422 (32.3)	1.219 (6.6)	15.630 (84.8)	**0.55 (0.49‐0.62)**	**<.001**	**0.59 (0.52‐0.67)**	**<.001**	**0.68 (0.59‐0.78)**	**<.001**
≥65	13.689 (24.0)	516 (3.8)	12.320 (90.0)	**0.30 (0.26‐0.34)**	**<.001**	**0.33 (0.28‐0.38)**	**<.001**	**0.40 (0.34‐0.46)**	**<.001**
Atopic dermatitis									
No	50.846 (90.8)	2.628 (66.3)	45.177 (94.3)	1	—	1	—	1	—
Yes	5.145 (9.2)	1.334 (33.7)	2.730 (5.7)	**8.40 (7.78‐9.07)**	**<.001**	**7.53 (6.96‐8.13)**	**<.001**	**7.28 (6.72‐7.88)**	**<.001**
Exposure to wet activities									
No	40.820 (75.4)	2.651 (66.9)	35.394 (76.8)	1	—	1	—	1	—
Yes	13.299 (24.6)	1.309 (33.1)	10.689 (23.2)	**1.64 (1.53‐1.75)**	**<.001**	**1.40 (1.31‐1.51)**	**<.001**	**1.33 (1.23‐1.44)**	**<.001**
Smoking									
Never	26.343 (47.0)	2.006 (49.1)	22.376 (46.9)	1	—	1	—	1	—
Former	20.521 (36.6)	1.299 (31.8)	17.608 (36.9)	**0.82 (0.77‐0.89)**	**<.001**	1.06 (0.98‐1.14)	0.15	1.07 (0.98‐1.16)	0.13
Current	9.454 (16.8)	783 (19.2)	7.905 (16.5)	**1.11 (1.01‐1.21)**	**0.02**	**1.13 (1.03‐1.23)**	**0.007**	1.07 (0.97‐1.18)	0.18
0.1‐7 cig/day	3.640 (6.5)	277 (6.8)	3.085 (6.5)	1.00 (0.88‐1.14)	0.98	0.98 (0.87‐1.13)	0.87	0.92 (0.80‐1.07)	0.28
≥8 cig/day	5.557 (9.9)	480 (11.8)	4.602 (9.7)	**1.16 (1.05‐1.29)**	**0.005**	**1.22 (1.09‐1.35)**	**<.001**	**1.15 (1.02‐1.29)**	**0.02**
Pack‐years									
<15 pack‐years	20.047 (36.7)	1.426 (35.8)	16.970 (36.5)	0.94 (0.87‐1.01)	0.07	1.04 (0.97‐1.12)	0.27	1.04 (0.96‐1.12)	0.38
≥15 pack‐years	8.271 (15.1)	551 (13.8)	7.099 (15.3)	**0.87 (0.79‐0.96)**	**0.004**	**1.25 (1.13‐1.38)**	**<.001**	**1.18 (1.06‐1.32)**	**0.004**
Alcohol consumption									
Nondrinker	11.122 (21.0)	879 (22.7)	9.281 (20.6)	1	—	1	—	1	—
≤1 drink/day	26.602 (50.2)	2.060 (53.1)	22.441 (49.9)	0.97 (0.89‐1.05)	0.46	1.03 (0.94‐1.12)	0.56	1.07 (0.97‐1.17)	0.18
>1‐2 drinks/day	11.186 (21.1)	688 (17.8)	9.657 (21.5)	**0.75 (0.68‐0.83)**	**<.001**	0.95 (0.85‐1.06)	0.33	0.99 (0.88‐1.11)	0.85
>2 drinks/day	4.042 (7.6)	249 (6.4)	3.580 (8.0)	**0.73 (0.64‐0.85)**	**<.001**	1.01 (0.86‐1.17)	0.94	1.00 (0.85‐1.18)	0.99
Stress									
LTE									
0	25.042 (44.7)	1.693 (41.5)	21.520 (45.2)	1	—	1	—	1	—
1	15.665 (28.0)	1.177 (28.8)	13.238 (27.8)	**1.13 (1.05‐1.22)**	**0.002**	**1.14 (1.05‐1.23)**	**0.001**	**1.10 (1.01‐1.19)**	**0.03**
2	8.905 (15.9)	704 (17.2)	7.489 (15.7)	**1.20 (1.09‐1.31)**	**<.001**	**1.20 (1.09‐1.32)**	**<.001**	**1.16 (1.05‐1.28)**	**0.004**
≥3	6.424 (11.5)	509 (12.5)	5.373 (11.3)	**1.20 (1.09‐1.34)**	**<.001**	**1.21 (1.09‐1.35)**	**<.001**	**1.15 (1.03‐1.29)**	**0.02**
LDI									
0	12.708 (22.7)	603 (14.8)	11.342 (23.8)	1	—	1	—	1	—
1‐2	21.989 (39.3)	1.504 (36.9)	18.869 (39.6)	**1.50 (1.36‐1.65)**	**<.001**	**1.29 (1.17‐1.43)**	**<.001**	**1.22 (1.09‐1.35)**	**<.001**
3‐4	12.498 (22.3)	1.085 (26.6)	10.328 (21.7)	**1.98 (1.78‐2.19)**	**<.001**	**1.54 (1.38‐1.71)**	**<.001**	**1.39 (1.24‐1.56)**	**<.001**
≥5	8.825 (15.8)	889 (21.8)	7.069 (14.8)	**2.37 (2.12‐2.63)**	**<.001**	**1.69 (1.51‐1.89)**	**<.001**	**1.42 (1.25‐1.60)**	**<.001**
BMI (kg/m^2^)									
<18.5	449 (0.8)	41 (1.0)	367 (0.8)	1.26 (0.91‐1.75)	0.16	0.98 (0.70‐1.36)	0.89	1.00 (0.70‐1.43)	1.00
18.5‐25	26.291 (46.1)	1.970 (47.4)	22.258 (45.9)	1	—	1	—	1	—
25‐30	22.155 (38.9)	1.498 (36.0)	19.042 (39.3)	**0.89 (0.83‐0.95)**	**<.001**	**1.09 (1.02‐1.17)**	**0.02**	1.05 (0.97‐1.14)	0.22
>30	8.132 (14.3)	649 (15.6)	6.813 (14.1)	1.08 (0.98‐1.18)	0.12	**1.23 (1.12‐1.35)**	**<.001**	**1.15 (1.04‐1.28)**	**0.007**
Waist circumference (cm)									
≤80	13.188 (23.1)	1.130 (27.2)	10.919 (22.5)	1	—	1	—	1	—
>80‐90	17.593 (30.9)	1.279 (30.8)	14.911 (30.8)	**0.83 (0.76‐0.90)**	**<.001**	1.05 (0.96‐1.14)	0.29	1.06 (0.97‐1.17)	0.22
>90‐100	15.765 (27.6)	1.055 (25.4)	13.594 (28.0)	**0.75 (0.69‐0.82)**	**<.001**	**1.17 (1.07‐1.29)**	**0.001**	**1.17 (1.05‐1.30)**	**0.004**
>100‐110	7.439 (13.0)	477 (11.5)	6.452 (13.3)	**0.71 (0.64‐0.80)**	**<.001**	**1.20 (1.06‐1.35)**	**0.003**	**1.15 (1.01‐1.31)**	**0.03**
>110	3.042 (5.3)	217 (5.2)	2.605 (5.4)	**0.81 (0.69‐0.94)**	**0.005**	**1.30 (1.11‐1.52)**	**0.001**	**1.25 (1.05‐1.49)**	**0.01**
Physical activity (min/wk)									
No MVPA (0)	3.363 (6.4)	268 (6.9)	2.831 (6.4)	1	—	1	—	1	—
MVPA‐T1 (>0‐240)	16.403 (31.3)	1.371 (35.5)	13.710 (30.8)	1.06 (0.92‐1.21)	0.43	1.00 (0.87‐1.15)	0.96	1.00 (0.87‐1.16)	0.97
MVPA‐T2 (>240‐725)	16.348 (31.2)	1.197 (31.0)	13.833 (31.0)	0.91 (0.80‐1.05)	0.20	0.98 (0.85‐1.13)	0.79	0.96 (0.83‐1.12)	0.59
MVPA‐T3 (>725)	16.356 (31.2)	1.024 (26.5)	14.199 (31.9)	**0.76 (0.66‐0.88)**	**<.001**	1.08 (0.93‐1.25)	0.30	1.01 (0.87‐1.19)	0.86
No VPA (0)	8.127 (15.5)	609 (15.8)	6.884 (15.4)	1	—	1	—	1	—
VPA‐T1 (>0‐120)	15.857 (30.2)	1.267 (32.8)	13.329 (29.9)	1.07 (0.97‐1.19)	0.16	1.02 (0.92‐1.13)	0.68	1.00 (0.89‐1.11)	0.97
VPA‐T2 (>120‐290)	13.715 (26.1)	1.025 (26.6)	11.657 (26.2)	0.99 (0.90‐1.10)	0.91	0.98 (0.88‐1.09)	0.66	0.98 (0.87‐1.10)	0.69
VPA‐T3 (>290)	14.771 (28.2)	959 (24.8)	12.703 (28.5)	**0.85 (0.77‐0.95)**	**0.003**	0.95 (0.85‐1.06)	0.34	0.91 (0.81‐1.02)	0.11
Diet									
Vegetarian/vegan									
No	54.752 (97.9)	3.970 (97.5)	46.567 (98.0)	1	—	1	—	1	—
Yes	1.185 (2.1)	103 (2.5)	974 (2.0)	**1.24 (1.01‐1.52)**	**0.04**	1.14 (0.92‐1.40)	0.23	1.15 (0.91‐1.44)	0.24
LLDS (quintiles)									
Q1: 0‐18	7.568 (15.2)	623 (17.1)	6.430 (15.2)	1	—	1	—	1	—
Q2: 19‐22	10.359 (20.8)	801 (22.0)	8.828 (20.8)	0.94 (0.84‐1.05)	0.24	0.99 (0.89‐1.11)	0.90	1.02 (0.90‐1.15)	0.79
Q3: 23‐25	9.498 (19.1)	698 (19.1)	8.060 (19.0)	0.89 (0.80‐1.00)	0.05	0.99 (0.88‐1.11)	0.81	0.99 (0.87‐1.12)	0.86
Q4: 26‐29	11.378 (22.8)	807 (22.1)	9.609 (22.7)	**0.87 (0.78‐0.97)**	**0.01**	0.98 (0.88‐1.10)	0.78	1.01 (0.89‐1.14)	0.92
Q5: 30‐48	11.035 (22.1)	717 (19.7)	9.437 (22.3)	**0.78 (0.70‐0.88)**	**<.001**	0.92 (0.82‐1.04)	0.19	0.95 (0.84‐1.08)	0.45
Sleep (h)									
≤5	652 (1.2)	38 (0.9)	564 (1.2)	0.77 (0.56‐1.08)	0.13	0.97 (0.70‐1.36)	0.87	0.91 (0.62‐1.31)	0.60
>5‐7	23.688 (42.1)	1.715 (41.6)	20.071 (42.0)	0.98 (0.92‐1.05)	0.59	**1.09 (1.02‐1.16)**	**0.01**	1.07 (0.99‐1.15)	0.07
>7‐9	31.169 (55.4)	2.307 (55.9)	26.518 (55.5)	1		1	—	1	—
>9	769 (1.4)	67 (1.6)	654 (1.4)	1.17 (0.91‐1.52)	0.21	1.09 (0.84‐1.41)	0.51	1.00 (0.74‐1.34)	0.97

*Note*: Univariate and multivariate analysis presented as odds ratios (ORs) with 95% confidence intervals (CIs). Participants with self‐reported hand eczema in the past year were compared to participants without hand eczema ever. *P*‐values <.05 are shown in bold. Data on age, HE, atopic dermatitis, and exposure to wet activities were part of the add‐on questionnaire; all other variables were included in the baseline assessment.

Abbreviations: BMI, body mass index; CI, confidence interval; cig, cigarettes; cm, centimeter; h, hour; HE, hand eczema; kg/m^2^, kilogram per square meter; LDI, Long‐term Difficulties Inventory; LLDS, LifeLines Diet Score; LTE, List of Threatening Experiences; min/wk, minutes/week; MVPA, moderate and vigorous physical activity; n, number; OR, odds ratio; Q, quintile; T, tertile; VPA, vigorous physical activity; y, year. For missing data please see online supplemental material S5.

^a^ Model 1: adjusted for age and sex.

^b^ Model 2: adjusted for: age, sex, atopic dermatitis, and exposure to wet activities.

^c^ For the categories HE in the past year and HE never, prevalence of HE in the past year and HE never, rather than proportions, for the respective age groups are shown.

In the univariate analysis a positive association between HE in the past year and being female, having AD, and exposure to wet activities was found. Age showed a negative association with HE in the past year. Regarding lifestyle factors, there was a positive association between HE in the past year and smoking, stress, and a vegetarian/vegan diet at baseline. A negative association was found between HE and former smokers, a history of ≥15 pack years, alcohol consumption, BMI (25‐30 kg/m^2^), waist circumference, the LLDS score, and physical activity at baseline.

Associations between HE and age, sex, AD, and exposure to wet activities were similar in all adjusted models compared with the univariate analysis. When adjusting for age and sex (model 1), HE in the past year showed a positive association with smoking, a history of ≥15 pack years, stress, overweight and obesity, a waist circumference of >90 cm and sleeping 5‐7 hours per 24 hours at baseline.

When also adjusting for AD and exposure to wet activities in addition to age and sex (model 2), HE in the past year was more common in individuals reporting smoking ≥8 cigarettes/day, or a smoking history of ≥15 pack years at baseline. Furthermore, positive associations between HE and a BMI >30, a waist circumference of >90 cm, and individuals reporting more stress according to both the LTE and LDI at baseline were found. No association between HE and amount of sleep at baseline was found. In addition, no statistically significant associations for former smoking, alcohol consumption, physical activity, and diet at baseline were found in either adjusted models.

When adjusting for age, sex, AD, exposure to wet activities, and all lifestyle factors (model 3: see online supplementary material S3) similar results regarding all variables were found compared to model 2. When replacing all categorized variables for the continuous variables (see online supplementary material  S4) similar directions of associations were found compared with the categorized variable.

## 4 DISCUSSION

HE in the past year was more common in individuals who reported smoking, chronic stress, obesity, or a higher waist circumference at baseline. No associations with other lifestyle factors were found. Associations between previously reported risk factors for HE as being female, a younger age, AD, and exposure to wet activities were confirmed in the current study.

Our results are partly in line with previous studies reporting the association between the occurrence of HE and several lifestyle factors. A cross‐sectional public health survey from Sweden, including 27 793 individuals, also found a positive association between HE in the past year, current smoking, stress, and obesity.^22^ However, it was reported that HE was less common among individuals reporting high physical exercise, which could not be confirmed in the current study. Although, results are hard to compare due to the different questions and tools used for measuring physical activity. In addition, in the previous study, physical exercise in leisure time and physical activity at work were categorized as two separate lifestyle factors. Physical activity at work showed a positive association with HE instead of the negative association with physical exercise. Another study in 2 688 participants of the National Health and Nutrition Examination Survey (NHANES) database reported that subjects who reported recent vigorous or moderate physical activities during leisure time were less likely to have active hand dermatitis.^23^ In the current study, both physical activity in leisure time and physical activity at work were included in the lifestyle factor physical activity. Exercising in leisure time might be inversely associated with HE, whereas physical activity at work, possibly in occupations with more irritant exposure to the hands, might contribute to the occurrence of HE. Moreover, it is possible that individuals with current HE avoid particular types of exercise, especially not mandatory physical activity in leisure time, due to the complaints of their HE. Another cross‐sectional study, among 1 870 health care workers with HE in the past year, also found a positive association between HE and obesity (BMI ≥30), and stress in a dose‐dependent manner. However, smoking was not associated with HE.^24^

It has been debated whether tobacco smoking is associated with HE, as previous studies have been pointing in different directions. A systematic review and a meta‐analysis reported the evidence of HE and smoking.^11^, ^12^ No association between smoking and the prevalence of HE was found in the meta‐analysis. However, due to a lack of numerical data to perform the meta‐analysis, this conclusion was based on only three studies, all conducted in the same country.^11^ On the other hand, another systematic review reported cautiously that smoking may cause an increased prevalence and severity of HE, especially in high‐risk occupations.^12^ Several other chronic inflammatory skin diseases are linked to smoking, such as AD,^25^ palmoplantar pustulosis,^26^ and psoriasis.^27^ Smoking has an immunomodulatory effect with elevated levels of immunoglobulin E (IgE), increased macrophage and dendritic cell activity, a release of pro‐inflammatory cytokines, and a favored activity of the Th2 pathway.^28^ In addition, nonimmunologic effects such as cutaneous vasoconstriction with delayed wound healing and chronic damages to the microcirculation might play a role in HE.^29^ Moreover, especially in HE, the direct toxic effects of holding tobacco products, or the direct effect of tobacco products causing allergic contact dermatitis, might also influence the course of HE.^30^ In the current study, a positive association between HE and smoking eight or more cigarettes per day was found, pointing in a direction of a positive dose‐dependent relation between HE and smoking. It is possible that due to different categorization of smoking habits in previous studies, often categorized as smoking yes/no, the effect of higher daily smoking amounts is not revealed. Another possible explanation might be that smoking does not have much influence on the occurrence of HE itself, but it might act as a catalyst in individuals already prone to develop HE, leading to a higher severity and/or a worse prognosis of HE. This is supported by a few studies. A prospective multicenter cohort study in 1 608 patients with occupational HE found an increased severity and a worse prognosis of HE in smokers.^31^ In another, questionnaire‐based cross‐sectional study a strong association between tobacco smoking and HE severity was reported.^32^ In addition, in a recent register‐based cohort study of 1 491 individuals with HE, current smoking was inversely associated with healing of HE.^33^

The positive association between HE in the past year and chronic stress found in the current study is in line with studies of Anveden et al. and Hamnerius et al.^22^, ^24^ However, the current study is the first to use validated instruments to investigate chronic stress. Previous studies investigating the association between stress and HE used just a single question reflecting the subjective experience of stress in the individual. It is known that having HE, especially severe HE, might cause stress as well. However, in this study a broad range of stressful life events is used to indicate the level of experienced stress, and only one question included health aspects. All other questions are not directly health related and might therefore better indicate stress beyond HE. This supports the direction of stress contributing to the occurrence of HE rather than HE causing stress. An explanation of the found positive association between occurrence of HE and stress, might be a link between the central nervous system (CNS) and the immune system represented by the hypothalamic‐pituitary‐adrenal (HPA) axis.^34^ Both emotional and psychological stress might lead to the releases of corticotropic‐releasing hormone, which activate the HPA leading to increased endogenous glucocorticoid, a modulation of the inflammatory response, and a decrease in epidermal lipid synthesis, antimicrobial defense, and barrier ability.^35^ In addition, chronic stress may induce an imbalance of T helper (Th) 1/Th2 responses in favor of the Th2‐mediated response, which can contribute to HE.^36^ Another possible reason could be that one of the main causes of HE is atopic HE, which can be triggered by stress as well.^37^

HE was associated with obesity and a higher waist circumference in the current study. Two previous studies have also reported the association between a BMI ≥30 kg/m^2^ and the occurrence of HE.^22^, ^24^ This is the first study including results on waist circumference in individuals with HE. Waist circumference might act as an indicator of central obesity, which is associated with increased visceral fat. Increased visceral fat acts as an endocrine organ that activates macrophages and releases pro‐inflammatory cytokines that might lead to immune dysregulation.^38^

In the current study no association between HE and alcohol consumption was found, which is in line with previous studies.^22^, ^39^, ^40^, ^41^ Only one other study investigated HE and a vegetarian/vegan diet in 6 095 adolescents, and also no association was found.^42^ The association between diet quality and HE has not been studied before. However, the variety of definitions of quality are almost infinite; therefore, especially in the absence of extensive research conducted in HE, no directions in associations regarding HE and diet can be made yet.

In the current study, responders reported a healthier lifestyle compared with nonresponders. In addition, the 1‐year prevalence of HE might be slightly overestimated because nonresponders were more likely to be male and less likely to have skin diseases than responders. Based on the selective nonresponse bias, some of the associations might be missed based on the higher prevalence of healthy lifestyle factors and HE in the subjects included in the present analysis.

One of the limitations of the current study is the difference in moment of data collection of lifestyle factors and data regarding HE. Because this study is a cross‐sectional add‐on questionnaire within a cohort study, data regarding exposure (lifestyle factors) were collected at baseline between 2006 and 2013 and questions regarding HE were collected in 2020. Therefore, temporality of lifestyle factors, especially those factors that might change easily over time such as smoking habits and diet, is important to consider. The strengths of this study are the large sample size, the possibility of performing a nonresponder analysis, and the use of a validated tool to measure physical activity and chronic stress. Even though the SQUASH,^19^ LTE^16^, ^18^ and LDI^18^ are validated instruments to measure physical activity and stress in population‐based cohort studies, no interpretability is currently available. Categorization was therefore done arbitrary, where an influence on HE seemed to be plausible. In addition, when replacing the categorized variables for the original continuous variables, the directions of found associations were similar. Due to the explanatory approach of this study, *P*‐values were not adjusted for multiple testing. However, almost all found positive associations were based on *P*‐values considerably lower than .05. A limitation of the current study is that data on HE was self‐reported and might therefore be incorrect in some cases. In addition, data on lifestyle factors could be deviated by socially acceptable answers. However, to data collection in the general population produces such large numbers, so the use of self‐reported data is often unavoidable. Another limitation is that no data on the subtyping of HE were available, whereas there is some evidence that different subtypes may have impacts different from lifestyle factors.^43^, ^44^, ^45^, ^46^

## 5 CONCLUSION

In conclusion, this study showed a positive association between HE in the past year, smoking, chronic stress, obesity, and waist circumference. Because personalized medicine is a subject of increased interest, and future health care is moving forward to a more individual approach, attention to lifestyle interventions such as reducing stress, losing weight, and quitting smoking to promote better overall health may be important to include when counseling patients with HE. However, to get a better understanding of the effect of lifestyle factors on HE, it is important that further studies also focus on the association between lifestyle factors and the severity and prognosis of HE rather than on occurrence alone.

## CONFLICT OF INTEREST

M.L.A. Schuttelaar received consultancy fees from Sanofi‐Genzyme and Regeneron Pharmaceuticals; and is an advisory board member for Sanofi‐Genzyme, Regeneron Pharmaceuticals, Pfizer, LEO Pharma, and Lilly. No other conflicts are reported.

## AUTHOR CONTRIBUTIONS

All authors have participated sufficiently to take public responsibility for appropriate portions of the work. Conceptualization: Marie L. A. Schuttelaar, Laura Loman (equal). Data curation: Laura Loman (lead). Formal analysis: Laura Loman (lead). Funding acquisition: Marie L. A. Schuttelaar (lead). Investigation: Marie L. A. Schuttelaar, Laura Loman (equal). Methodology: Marie L. A. Schuttelaar, Laura Loman (equal). Project administration: Laura Loman (lead). Resources: Marie L. A. Schuttelaar (lead). Software: N/A. Supervision: Marie L. A. Schuttelaar (lead). Validation: N/A. Visualization: Marie L. A. Schuttelaar, Laura Loman (equal). Writing original draft: Laura Loman (lead). Writing‐review & editing: Marie L. A. Schuttelaar (lead), Laura Loman (supporting).

## Supporting information


**Appendix S1**. Supplementary Information.

## Data Availability

The data that support the findings of this study are available from the corresponding author upon reasonable request.
